# Review on camel production and marketing status in Ethiopia

**DOI:** 10.1186/s13570-022-00248-2

**Published:** 2022-09-12

**Authors:** Doyo Kena

**Affiliations:** College of Agriculture and Natural Resource, Jinka University, Jinka, Ethiopia

**Keywords:** Pastoralism, Arid and semi-arid areas, Camel export, Camel populations

## Abstract

Pastoralism has been the most productive livelihood option in the dryland of the Horn of Africa although recently its sustainability is becoming challenging. Camel is a livestock species uniquely adapted to the arid and semi-arid areas of the region. Camels are predominantly reared in the drier areas of Ethiopia such as Afar, Ethiopian Somali and the eastern and southern parts of Oromia region. This review is aimed at reviewing the camel population, marketing status, challenges and opportunities related to camel production and marketing in Ethiopia. Official reports on the camel population (1.42 million) underestimate the number of camel populations in Ethiopia while different research reports a higher figure of the camel population up to 4.8 million. However, each report indicated an increasing trend in the camel population. Camel is being adopted by different pastoral groups, in which camel rearing was less customary. The economic importance of the camel over other livestock species is immense, particularly during the harsh seasons due to less decline in its prices and the camel is the most expensive in both pastoral and agro-pastoral areas when compared to other livestock. Camel plays an important role in revenue generation, contributing to the earnings from export. Formal camel export status has shown a flat trend and informal export outweighed the formal one. Despite its ecological and economic importance, the camel has been neglected by researchers and the Ethiopian government. Poor market infrastructure, lack of market information, lack of market-oriented production system, the export ban by many countries and the inconvenience of an export regulatory institutional setting are among the major constraints of camel marketing in Ethiopia. Therefore, policy and development interventions are demanded that recognize the social, economic and ecological importance of camels for pastoral communities and the national economy.

## Introduction

Sub-Saharan African dryland is known for its scarcity of rainfall, environmental aridity, climate variability and less favourability to crop cultivation, both ecologically and economically. This in turn creates the spaces for livestock rearing due to their capability to better adapt to the harsh environment than other natural resource-based activities. Pastoralism has been the most productive livelihood option in the dryland of the Horn of Africa. However, pastoralism sustainability in the dryland area is becoming challenging especially in the Horn of Africa. According to Fratkin and Mearns (2003), pastoralists are said to be sustainable when maintaining livestock productivity, defending their rights and access to water and grazing resources and ensuring political and economic security.

Ethiopia has agro-ecological zones that allowed the existence of many livestock species and created a suitable environment for livestock production. Dryland covers the larger (60%) part of the Ethiopian land mass (Fre and Tesfagergis 2013). The direct contribution of the livestock sector to total output is estimated to be 17% of GDP and 39% of the agricultural GDP. The contributions increase to 21% of the national GDP and 49% of the agricultural GDP, when livestock and its product processing and marketing (35.6 billion) are considered. If the indirect contributions in organic fertilizer and traction (37.8 billion) are considered, the contribution of livestock to the GDP will rise to 25.3% (Shapiro et al. 2017). The contribution of the different livestock species to the total production is about 81.2% from cattle, 6.3% from camels, 7.9% from goats and 4.6% from sheep (CSA 2008).

Camel is one of the livestock species uniquely adapted to arid and semi-arid areas of the world. Arid lowlands of Eastern Africa namely, Somalia, Sudan, Ethiopia, Kenya and Djibouti, are mainly known for camel rearing (Dejene 2015). In Ethiopia, camels are predominantly kept in the pastoral and agro-pastoral production systems. Only few male camels are to be found in the mixed crop-livestock system (Mirkena et al. 2018). According to the Central Statistical Agency (2018), the camel population of Ethiopia is estimated to be above 1.42 billion that set the country sixth in Africa in camel population. This estimate is criticized by different reports that it underestimates the camel population in Ethiopia. For example, according to Wako (2015), the total camel population in Borana Zone in 2012 was around 119,223 which is by far greater than an estimate of the Central Statistical Agency in 2013 (62,789) (CSA, 2013).

Camel production is a major source of livelihood for the pastoralists in the arid and semi-arid lands (Kagunyu and Wanjohi 2014). Camels are a source of food, cash income and transport means and have significant cultural functions to pastoral communities dominating in the ASALs (Guliye et al. 2007; Mahmoud 2010). Camels play diverse roles in pastoralist's livelihood serving as building of assets, insurance against unexpected events, having spiritual and social values, traction and movement of goods, food supply and income (Ali et al. 2004).

Drier areas of the country like Afar, Ethiopian Somali and the eastern and southern parts of Oromia region contain the majority of the camels. According to Sisay and Awoke (2015), annual camel milk production in Ethiopia is estimated to be 170,000 tons which ranked the country fourth next to Somali, Kenya and Mali in Africa in milk production. The economic viability of camels in arid lands is assured by their comparative advantages in their ability to adapt and remain productive under harsh climatic conditions, more than other livestock (Noor et al. 2013).

Despite the enormous contributions of camels to the socio-economic life of the pastoral and agro-pastoral communities, serving as a source of food, income generation and transportation means up to ritual ceremonies of pastoral communities, minor emphasis has been given to camels and livestock in general on Ethiopian policy agenda and development prospects. Given the socio-economic importance of camels to the pastoral communities and the country, it is worthy to review the status of camel production and marketing. Therefore, this review paper focuses on the camel production and marketing status as well as sheds light on challenges and opportunities in the production and marketing of camels in Ethiopia.

## Camel population and distribution in Ethiopia

The camel (*Camelus dromedarius*) is an important livestock species uniquely adapted to arid and semi-arid environments (Zeleke 2017). Camel adaptation makes them survive rainless seasons on the scantiest feed and exist in areas where other livestock species cannot survive (Kagunyu and Wanjohi 2014). In the world, camel production is reported in 46 countries, with both dromedary and Bactrian (Faye 2020). In 26 African countries, only dromedary (one humped) camels are reared. A larger proportion of the world’s camel herd is reared in the drier area of Eastern Africa. The world camel populations by 2018 were about 38.5 million heads (FAOSTAT 2020). More than 80% of domesticated camels inhabit Africa, with 60% (11.8 million heads) in the Eastern African countries like Sudan, Somalia, Ethiopia and Kenya (Faye 2015).

Ethiopia shares numerous camel populations from the region, and the major ethnic groups owning camels in Ethiopia are the Beja, Rashaida, Afar, Somali, Karayu and Borana. In most camel-rearing societies, camel milk is mainly consumed in its raw state without being subjected to any sort of processing treatment (Sisay and Awoke 2015). According to Tefera and Abebe (2012), most of these pastoral groups live across different valleys: Kunama reside in the Tekeze valley, Irobe/Saho in the Alitena valley, Raya-Kobo in the Raya valley, the Afar in the Awash valley, Somali in the Wabishebele valley and the Borena in the Genalle valley, making the area from northeast to southeast the camel belt of Ethiopia.

An estimate of Ethiopian camel populations varies in different reports. Despite the variations in estimates, reports indicated increasing trends of the camel population in Ethiopia and camel production is being adopted by different pastoral groups, in which camel rearing was less customary (Coppock 2013; Mirkena et al. 2018; Yosef et al. 2013). Yosef et al. (2013) highlighted that camel population increment over the past 20 years lies between 10 and 25% in Gode, Jijiga, Shinille, Mille and Amibara while the increment climbed up to 200% in the Borana pastoralist zone. The camel population shows an increasing trend because of the combined effects of pastoralist needs and the impact of climate change. Coppock (2013) also noted largest gains in the percentage of camel since the mid‐1980s while cattle decreased from 92 to about 78% of TLUs.

According to Farah et al. (2004), Ethiopia’s camels’ herd has the third rank in the world after Somalia and Sudan. However, the Central Statistical Agency (2018) estimated the camel population of Ethiopia to be 1.42 million that set the country sixth in Africa in the camel population. This very low official figure for the camel population is reported as above 4.5 million heads based on several more reliable and recent surveys for the Afar, Somali and Borana regions (Shapiro et al. 2017). A report by Behnke (2010) also was consistent with the latter figure, presenting the camel population in Ethiopia to be around 4.8 million. Furthermore, reports by Zeleke (2017) reveal that the former figure by the Central Statistical Agency corresponds to the camel population only in sedentary parts of the country which grew from 247 thousand to 1.21 million with an average increment of 10% from 1996 to 2016. A study made by Faye (2020) reported that, this irregularity have linkage with lack of security in Somalia which led to the influx of pastoralists in some parts of Ethiopia and Kenya.

**Fig. 1:**
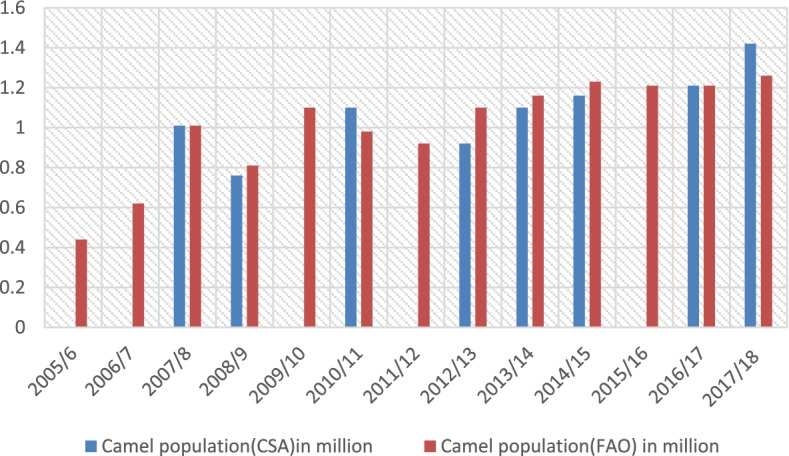
Trend of the camel population in Ethiopia by FAO and CSA. Source: author computation from FAO STAT and CSA

The trend of the camel population as to the CSA report, in Fig. 1, indicates rough increments of the camel population from 2008 to 2018 although there is fluctuation in some years. The trend indicated that the camel population declined below 1 million by 2009 and 2013 and after 2013 there is quite an increment in the camel population up to 1.42 million in 2017/2018.

The camel population status by FAO STAT is more or less similar to the Central Statistical Agency reports. Accordingly, FAO reports show that the camel population shows a fluctuating pattern from 2008 to 2012 being some time below one million in 2008, 2010 and 2012. From 2013 on, the Ethiopian camel population shows increasing trends. A report by Faye (2020) categorized Ethiopia under the countries with slight growth in camel population.

## Economic importance of the camel in Ethiopia

Camels’ contribution to the national economy and pastoralist livelihood is irreplaceable in Ethiopia. The economy of pastoralist communities relies mainly on livestock resources from which camels play a significant role. In camel-rearing African countries, there is a difference in proportions of the camel to their national livestock resources as well as its role in the national economy. According to Faye (2020), Somalia Labeled as the country with an extremely large proportion of camels (more than 35% of TLU of Somalian TLU is camel) and Djibouti is under countries with a high proportion of camel livestock (15–35% of TLU), from the Horn of African countries, while Sudan and Kenya fall under categories with a medium proportion of camel to livestock (5–15% of TLU). Ethiopia, which is known as the livestock tower of Africa, is categorized under countries with lower camel proportion (1–5% of TLU).

Despite the lower proportion of camels in the country and their role in the national economy, the export earning is very much exciting and encouraging. Economic contributions of camels to the pastoral households’ income increase recently when compared to other livestock in the pastoral lowland of the country. Yosef et al. (2013) and Bekele et al. (2008) noted that increased aridity in Borana Zone shifted the principal stock gradually from cattle combined with small stock to camels combined with small stock. Those reports also indicated that the social status of households increased with the increasing camel numbers in recent years. This shows the increased value given by the community to camels over other livestock species.

According to reports of Shapiro et al. (2017), camels are the second most expensive livestock per TLU next to poultry in agro-pastoral areas and the most expensive livestock per head in Ethiopia. But in pastoral areas, it is the most expensive livestock on both bases (per TLU and per head). If converted to TLUs, small ruminants and poultry seem to generate a higher return per TLU than cattle (Table 1).

**Table 1:** Livestock price information in pastoral and agro-pastoral areas of Ethiopia

Types of livestock	Pastoral area		Agro-pastoral	
	Price per head	Price per TLU	Price per head	Price per TLU
Cattle	767	767	1001	1001
Camel	5861	4186	5872	4194
Goat	263	2,630	264	2640
Sheep	175	1750	168	1680
Poultry	-	-	583	58,300

Source: Shapiro et al. (2017)

The comparative economic importance of the camels over other livestock species is immense during the harsh season due to a little decline in their prices. During prolonged dry seasons and droughts, prices for camels decline relatively little compared with those of other animal species. For example, in northern Kenya, camels’ price declines by 4–12% during the drought, while that of cattle drops by more than 60%. Sheep also experience a greater decline in price up to 40% or more during dry seasons, but prices for goats are less volatile, with about a 20% decline in the dry seasons (Barrett et al. 2003).

Moreover, due to both increased local demand and growing demand for camels and goats on international markets (that is, the Middle East and North Africa), camel prices have grown considerably faster than prices for other animal species, according to the analysis of the livestock market information system data for Kenya and Ethiopia (Aklilu and Catley 2011). Aklilu and Catley (2011) reported that average camel prices in Ethiopia have doubled in just the past 2 years, before the time of their report, due to increasing camel export to Sudan and then eventually to Egypt.

## Camel marketing status in Ethiopia

Livestock is an important contributor to export earnings (live animal exports), covering nearly about 8% (USD 211 million) of the USD 2.75 billion export earning achieved in 2011 (AGP-LMD (Agricultural Growth Project-Livestock Market Development) 2013). Ethiopia is exporting meat and live animals to different countries. Live animal exports contributed 70% of the earnings of animal export and the remaining share was that of meat and hide (USAID 2013). The United Arab Emirates is the largest importer of meat, buying 50% of the total meat exported followed by the Kingdom of Saudi Arabia with 30% (Trade bulletin 2011). Cattle accounted for 46% of total exported livestock, while sheep, camels and goats accounted for 35%, 13% and 6% respectively. In terms of revenue generation, camels contributed 23% to the total earning from livestock export (Trade bulletin 2011; USAID 2013).

Camel is one of the most traded livestock across borderlands of Ethiopia besides cattle, goats and sheep (Tesfaye and Amaha 2018). (Tesfaye and Amaha 2018) reported an annual estimate of about 10,000 camels being sold to inside and/or to border markets of Ethiopia. Annual reports of the Central Statistical Agency indicated that recently the live camel sale in Ethiopia is showing an increasing pattern with a slight change in slaughter within the country. This is because, unlike ruminant animals, consumption of camel meat is not that much common except in pastoral areas. Thus, the camel value chain is mainly the live camel value chain wherein camels are exported. Table 2 indicates that the annual sale of live camels was increasing after 2013/2014. The report of the Central Statistical Agency indicated that the amount of camel slaughter showed a decreasing pattern, and its volume is small due to limited consumption in certain parts of the country.

**Table 2:** Live camel sale and domestic slaughter in Ethiopia

Year	Slaughter	Total sales	Slaughter rate
2007/2008	7667	33,283	0.230358
2008/2009	6734	55,120	0.12217
2010/2011	7910	59,910	0.132031
2012/2013	5596	64,232	0.087122
2013/2014	2082	39,958	0.052105
2014/2015	4596	44,783	0.102628
2016/2017	3719	56,972	0.065278
2017/2018	6742	81,364	0.082862

Source: Central Statistical Agency

**Fig. 2:**
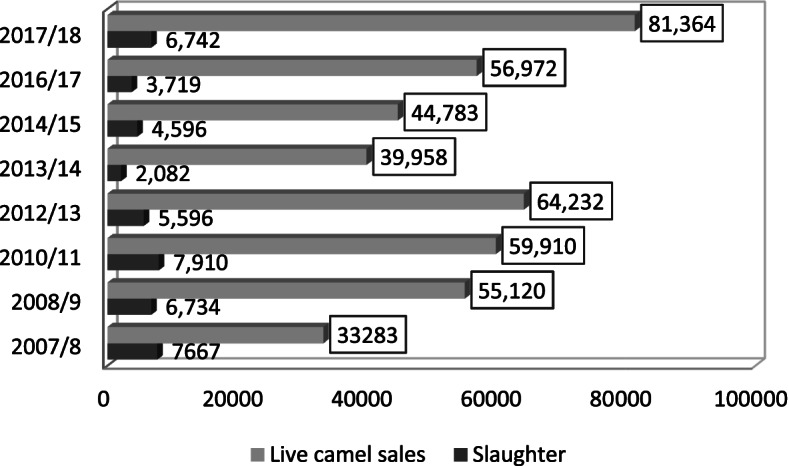
Live camel sale and domestic slaughter in Ethiopia. Source: author computation the Central Statistical Agency

According to data in Table 2 and Fig. 2, the camel slaughter rate in Ethiopia is ranging from 5.2 to 23% with an increasing trend after 2010. The highest slaughter rate was recorded in 2007/2008 which was about 23% while the average slaughter rate was 10.8% which is higher than the African average (7%) and regional or east African average (4.3%) (Kadim et al. 2013).

Estimates of the volume and value of cross‐border livestock exports vary by sources. For example, estimates of 2009 for the annual livestock exports from Ethiopia to Djibouti, Somaliland, Somalia and Kenya are 350,000 cattle, 1,100,000 small ruminants and 125,000 camels, with an estimated value of between US$250 million and US$300 million (SPS‐LMM 2009). A large share of Ethiopian formal live animal exports pass through the quarantine centre in Djibouti, and so are re‐exported. In 2008–2009, Ethiopia’s formal exports to or through Djibouti consisted of 103,010 cattle, 137,576 sheep, 79,349 camels and 11,319 goats. The informal trade volume from Ethiopia to Djibouti could be similar or higher, except for camels (Aklilu and Catley 2010).

The two major live camel sub-chains exporting camels to the Middle East and North African countries are the formal and informal live camel export sub-chains. Somali’s camel supply during 2009–2010 was around 74,000 heads to the Ethiopian formal camel export trade. Around 80% of the camels sold at Babile market (in Oromia region) originate from Liban, Afder, Fik, Kebridehar and Gode in Somali Region, because of better market opportunities than in southern Somalia. Similarly, most of the camels sold in Moyale, and to some extent in Negele Borana markets, originate from northern Kenya and southern Somalia (Aklilu and Catley 2010). Another formal camel sub-chain is the one in which animals are mainly exported through Metema to Sudan. Animals are transported from the other end of the country, as far as Moyale, on the border with Kenya (and sometimes from Kenya) to Metema with a stopover at Adama to fulfil the quarantine requirements in order to get export permits. About 36% of the total volume of animals entering the camel value chain passes through this sub-chain (Farmer 2010).

Table 3 indicates that there is fluctuation in both the number of camels exported and export value from 2010 to 2015. However, after 2016, data indicate a decreasing amount of export both in head and revenue generated from sale. This may be due to the reduction of the import of camels from the Horn of Africa to Saudi Arabia after 2012/2013, because of the MERS-Cov outbreak (Younan et al., 2016).

**Table 3:** Ethiopian live camel exports through formal channels (thousand head)

Year	2010	2011	2012	2013	2014	2015	2016	2017	2018	2019
No. of camel (000)	74.2	54.3	151.3	68.61	42.41	48.76	28.27	12.35	-	-
Value (USD Mln)	32.3	27.3	59.7	48.11	33.96	37.64	20.63	7.03	3.52	10.08

Source: ERCA, export data cited in Mamo, 2019

From camels entering the camel value chain, 64% are exported informally. Informal live animal trade from eastern Ethiopia (Somali Region) to Somaliland represents the largest share of cross-border trade in terms of volume and value (Alemayehu and Ayalew 2013). The main reason for this informal export business is the inconvenience of fulfilling formal export requirements. Analysis of the costs and margins along the camel value chain shows the two sub-chains are similar in terms of margins obtained by the different actors.

**Fig. 3:**
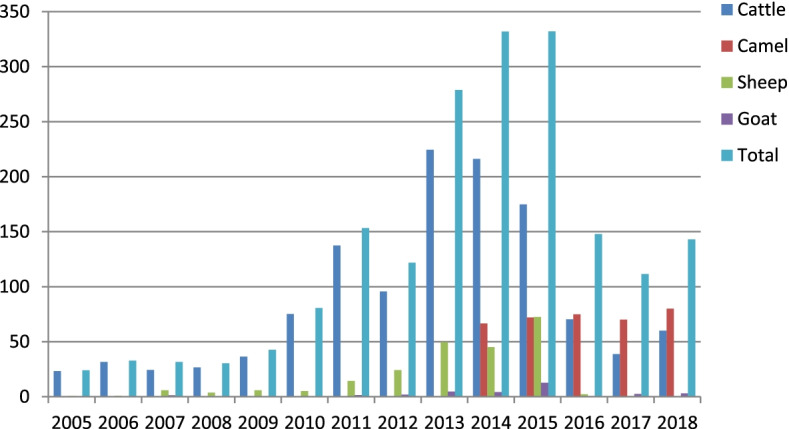
Data of export value of Ethiopian livestock (in millions USD) by FAO. Source: author computation from FAO STAT

According to data from FAO STAT, total livestock export showed a reduction after 2013 and it falls below 150 million after 2015 (Fig. 3). The reduction is mainly due to a decline in cattle export. However, the case of the camel is the opposite and exported value increased from year 2013. Camel export value is greater than that of other livestock after 2016.

In Ethiopia, both Somali and Borana pastoral areas can be described as ‘high export areas’. Although informal cross‐border trade still dominates, increasing policy support to meat exports, and to a lesser extent live animal exports, has resulted in a growing formal export of meat and animals from Ethiopia. This trend has also been driven by high demands in the Middle East, Egypt and Sudan. Camel exports are becoming increasingly important for Ethiopia (Aklilu and Catley 2010).

## Camel marketing challenges in Ethiopia

Despite its ecological and economic importance and significant role in the life of the pastoral community, until lately, the animals were neglected by researchers and development planners in Ethiopia (Yusuf and Tafesse 2004; AU 2010). Particularly, regarding camel, little is known about its production and health problems compared to other livestock (Dejene 2015). Farm Africa (2002) has observed that camels have been neglected and underused for a long time in Africa. Dejene (2015) made similar observations that, despite the advantages the camel has over other domestic animals, it has been neglected, with most research efforts being directed to cattle and shoats, among others.

Camel marketing challenges are numerous in Ethiopia and they are sourced from production constraints and cultural taboos and of policy and strategies of the country. Widespread diseases, poor veterinary service and lack of knowledge about camel production and treatments are among the major constraint related with camel production (Dejene 2015). This in turn had a tremendous effect on camel marketing particularly on the camel export market because until 2005 Ethiopian livestock export was banned from the international market due to the Rinderpest disease (Future Agriculture 2014). Furthermore, camel production and productivities has been contrained by several factors like recurrent drought, over-grazing of rangeland and enclosure of pastoral range land which in turn become a bottleneck for camel marketing (Bekele et al. 2002; Farah et al. 2004; Birhanu and Beyene 2015).

Religious and cultural taboos that prohibit camel milk and meat consumption in different parts of the country affected the emphasis given to the sector in Ethiopia. Some religions and cultures consider the camel as a ‘beast’ with incomplete function and camel milk and meat as an inferior product. This had great effects on the demands of the camel and its product consumption (Berhanu and Beyene 2015). Restriction of camel product consumption by some cultures and religious teaching could have reduced camel and camel milk prices unless pressing demand of camel products by Arab countries (Dejene 2015).

With regard to the livestock export market, Ethiopia’s export bundle is generally subject to higher tariffs in both developing and developed countries. In a sample of 114 countries (for which tariff information is available at the six digits of the HS), 87 countries impose a higher tariff on products exported by Ethiopia than on products on exports by other countries in the sample. Non-tariff barriers also set a serious problem for Ethiopian export. Sanitary and phyto-sanitary requirements in QUAD markets, for these products, are costly to meet when technically possible (USAID 2010).

Another most important challenge of camel marketing is its lack of a market-oriented production system and improper infrastructural market development which had an effect on the quality and consistent supply of camels to the market (Pavanello 2010). Solomon et al. (2003) reported that knowledge on livestock market structure, performance and price is poor and inadequate for designing policies and institutions to overcome perceived problems in the marketing system. Pastoralist take their livestock particularly camel to market place without prior marketing information, and it is just to satisfy their capital need or to cull the older or other camels not required for reproduction. There was no separate market place and day for marketing camels other than the one employed for other species of livestock. The long trekking distances to markets are a major constraint to livestock marketing. Poor and uneven access to market information is a well-known constraint to livestock trade in Ethiopia (Adugna 2006; Pavanello 2010).

Inconsistency of the sectors that oversee livestock marketing and other related issue is one of the greatest challenges which make the livestock subsector less productive and uncompetitive. Under the current structure, the responsibility for livestock development is diffused throughout various government ministries and authorities. Furthermore, the responsible department for livestock development at the national level has been under different institutions and the coordination between federal and regional levels is not fully clear (FAO 2015).

In addition to these internal challenges, Ethiopian livestock and meat exporters face stiff competition from Brazil, India, Pakistan, Australia and New Zealand. Brazilian beef is price competitive because of low production costs, while India has a ready-made market, catering primarily to the large non-resident Indian population in the Gulf for its beef (buffalo meat) exports. Australia, which exports primarily sheep meat to the Middle East, has an aggressive marketing campaign through its regional marketing office and targets the higher end of the market. Faced with such competitors, Ethiopian meat exporters find it difficult to compete on price or quality (Farmer 2010).

## Camel production and marketing opportunities in Ethiopia

Live animal exports are high, with an estimated 1.6 million livestock, and the Ethiopian government is committed to supporting meat exports (Farmer 2010). Besides government commitments in line with export, within country, there is engendered demand for livestock products due to rapid urbanization, extensive population growth, emerging trends in commercialization and change in the living standard of the societies in Ethiopia which are good opportunities also for camel and camel milk marketing in the future (Mebrahtu et al. 2017).

There is a logical economic link between pastoral mobility, efficient production and livestock exports. Although Ethiopian policies and development strategies lagged behind in recognizing this connection, this link has been recognized by regional economic bodies, such as COMESA and IGAD, and is supported in their emerging policy frameworks for pastoral areas (Aklilu and Catley 2010). Similarly, the new African Union Policy Framework for Pastoralism in Africa supports pastoral mobility and emphasizes the adaptive nature of pastoralism and its ecological and economic logic (African Union 2010). Ethiopia agreed to abide and work with these regional organizations such as COMESA and IGAD and currently agreed to implement African free movement (trade) without a visa which in turn enforces the country to obey the common policy and strategies set by these organizations.

Camel has both production and marketing advantages over other livestock species. Encroachments of grassland by bushes and other trees favour browser production than grazers. Due to increasing bush encroachments, it seems logical to rely more on browsing animals as the rangeland is often covered by bushes and trees and make economic use of them. Therefore, this has made the production environment more conducive to browsing goats and camels than grazing cattle (Gemtessa et al. 2007).

Particularly in pastoral lowland, the reduction of cattle productivities particularly milk has increased the attractiveness of camels among herders in Ethiopia since camels produce more milk than cows (Birhanu and Beyene 2015). During prolonged dry seasons and droughts, prices for camels decline relatively little compared with those of other animal species (Little and McPeak 2014). According to Sisay and Awoke (2015), in some pastoral areas, camel milk is preferred to that of cows because cow milk tends to make people fat while camel milk gives strength, endurance and stamina, an attribute that pastoralists need in order to pursue a nomadic lifestyle. These in turn maintain camel demand in the area. High drought tolerance of the camel relative to cattle together with the rapidly escalating price of camels is a further incentive for camel production and marketing in pastoral areas of Ethiopia (Aklilu and Catley 2010; Desta 2011).

Generally, Ethiopia’s lowland breeds of cattle, sheep, goats and camels are highly demanded by neighbouring countries as well as the strategic livestock markets of the Middle East (Hurrissa and Eshetu 2003). Therefore, Ethiopia has some important comparative advantages in the Middle Eastern livestock and meat markets. Geographical proximity to Egypt and the Gulf makes both live animal exports and chilled meat exports possible (Farmer 2010). The relatively huge number of livestock resources, proximity to the export markets, liberalization of the economy and supports and attentions given by the government to export trade give the country comparative advantages in livestock trade (Shibru 2017).

## Conclusion

Rainfall scarcity and environmental aridity in the dryland of East Africa make the area suitable for pastoralism than any other livelihood option because of its economic profitability and environmental adaptability. In Ethiopia, camel production obtained exclusive emphasis by pastoralist communities over other livestock species particularly cattle because of its unique adaptability to drought and harsh conditions. Despite this fact, government policy put lesser emphasis generally on livestock production and camel production particularly. Ethiopia shares numerous camel populations with the Horn of African countries. Camels are many things for the pastoral communities serving as a source of food through direct consumption, income generation, transportation means, wealth accumulation, ritual activities and up to draught power in some ethnic groups. Ethiopia is known for camel rearing for a long time and its camel population status increased from time to time although official figures are far below many other reports. The estimate ranges between 1.42 million and 4.8 million. This review indicated that the official report on the camel population by the Central Statistical Agency and FAO underestimated the camel population of the country.

Despite the lower proportion of camel in the country’s livestock resource and national economy, export earning is very much exciting and encouraging. Economic contributions of the camels to the pastoral households’ income increase from time to time when compared to other livestock in the pastoral lowland of the country. Camel rearing is becoming the most expensive both per head and TLU in pastoral areas of Ethiopia. Camel production is constrained by problems such as recurrent drought, environmental degradation, disease, poor veterinary service and lack of knowledge about camel production and treatment. Marketing challenges are lack of market-oriented production system and improper infrastructural market development, trade ban by many countries, lack of marketing information, inconvenience-related export regulatory systems, stiff competition with other countries and many other factors. Despite the challenges, there are increasing opportunities of camel production and marketing such as increasing aridity, increasing demand of camel products both from domestic and abroad, some reform in policy setting pushed by regional organizations and proximity to gulf countries. Therefore, policy and development intervention that recognizes the social, economic and ecological importance of camel for pastoral communities and nationwide is demanded.

## Data Availability

Not applicable.
